# Recurrence of periodontitis and associated factors in previously treated periodontitis patients without maintenance follow-up

**DOI:** 10.34172/japid.2020.010

**Published:** 2020-06-16

**Authors:** Fazele Atarbashi-Moghadam, Mohammadreza Talebi, Farnaz Mohammadi, Soran Sijanivandi

**Affiliations:** ^1^Department of Periodontics, Dental School of Shahid Beheshti University of Medical Sciences, Tehran, Iran; ^2^Dental Research Center, Research Institute of Dental Sciences, Shahid Beheshti University of Medical Sciences, Tehran, Iran

**Keywords:** Gingivitis, Periodontitis, Periodontal maintenance, Supportive periodontal treatment

## Abstract

**Background:**

Preventive dentistry, including supportive periodontal therapy (SPT), is one of the most critical areas of attention. Despite SPT’s importance in the long-term success of periodontal treatment, the patients’ adherence to it is weak. The present study aimed to evaluate of periodontal disease’s recurrence rate and its related factors in periodontal patients without regular follow-up.

**Methods:**

A cross-sectional study was set in a specialized periodontics clinic in Tehran, Iran. Patients with periodontitis who completed periodontal therapy during 2005–2014 and did not adhere to the maintenance phase were evaluated. The periodontal history of the patients was updated. The previous diagnoses of patients according to their previous periodontal charts were revised by AAP 2015 criteria. Then, periodontal parameters were assessed, and current periodontal status was evaluated. Statistical analyses consisted of Fisher’s exact test, t-test, Man-Whitney test, and Kruskal-Wallis test. Spearman’s correlation coefficient was used to assess the relationship between factors and variables.

**Results:**

Fifty patients were evaluated, including 29 males and 21 females. There was a significant relationship between the initial diagnosis and recurrence rate of periodontitis (P=0.017). There was also a significant relationship between the recurrence of periodontitis and the years elapsed since the initial treatment (P=0.027, r = 0.353). Smoking significantly affected tooth loss (P=0.001).

**Conclusion:**

Patients with severe periodontitis need more attention to participate in supportive periodontal care. The patients must be aware of the disadvantages of neglecting this phase and be reminded of regular follow-up.

## Introduction

Supportive periodontal treatment (periodontal maintenance) is a phase of periodontal therapy in which the periodontal condition is monitored, and etiological factors are reduced or eliminated after the completion of periodontal treatment.^1,2^ In this phase, oral, dental, and periodontal conditions are evaluated, including radiographic assessment, and supragingival and subgingival plaque and calculus are removed, and oral hygiene instructions are reviewed and reinforced.^3,4^ This phase of periodontal therapy significantly affects the periodontal prognosis and tooth survival by reducing the recurrence rate and tooth loss.^1,5,6^ The maintenance intervals are planned for each patient according to their specific risk factors, such as smoking habits, systemic diseases (e.g., diabetes), age, poor oral hygiene, and pocket depth >6 mm.^2,7^ However, there are different recommendations for proper intervals for patient follow-up, ranging from several weeks up to even more than one year (e.g., 18 months).^5^

Despite the importance of the maintenance phase, lack of patients’ adherence to this program leads to problems.^5^ It is found that adherence to recall intervals in men is less than women, with the youth less than the middle-aged. Patients undergoing periodontal surgery had more compliance with the maintenance program.^8,9^ It is suggested that one of the main causes of irregular attendance in the maintenance program’s recall sessions is that patients prefer returning to their general dentists.^10^ Therefore, planning for patient referrals to their general dentists with a recommended strategy for long-term supportive periodontal care seems more practical.^11^ Considering the importance of this therapeutic phase and the patients’ unwillingness to participate in regular recall sessions, the present study aimed to investigate the recurrence rate of periodontal disease and its related factors in periodontal patients without regular follow-up.

## Methods

Patients with periodontitis who had been treated between 2006 and 2015 in a specialized periodontics clinic in Tehran, Iran, and completed the periodontal treatment but did not return for follow-up sessions of the maintenance program were recalled. Fifty patients participated in this study after signing written informed consent forms. This study conformed to the Declaration of Helsinki^12^ and was approved by the Ethics Committee of Shahid Beheshti University of Medical Sciences (code: IR.SBMU.RIDS.REC.1395.299).

Age, gender, history of systemic disease, and smoking habits of each participant were recorded. The periodontal examination was carried out by a periodontist using the Williams probe (Hu-Friedy, USA). Bleeding on probing (BOP), probing depth (PD) >4 mm, clinical attachment loss (CAL), furcation involvement (FI) according to Glickman classification,^13^ tooth mobility according to Miller’s classification,^14^ and O’Leary plaque index (PI) were recorded. Eventually, the diagnosis of the current periodontal status of each individual was reported based on AAP 2015,^15^ as gingivitis and periodontitis (mild, moderate, and severe). Participants without clinical symptoms of inflammation and with BOP less than 20% were considered as healthy.^16^ To prevent the results from being confounded, the previous diagnoses of patients according to their previous periodontal charts were modified by AAP 2015 criteria.^15^ The data underwent statistical analyses. Fisher exact test, t-test, Man-Whitney test, and Kruskal-Wallis test were used for statistical analyses. Spearman’s correlation coefficient was used to assess the relationship between factors and variables. P-value<0.005 was considered statistically significant.

## Results

Of 50 patients participating in this study, 29 were male, and 21 were female with an overall mean age of 53.5±9.546 years. The youngest and oldest participants had 36 and 79 years of age, respectively. The average years elapsed since their treatment was 6.96±1.653. The minimum years elapsed since the treatment was three, and the maximum was nine years. None of these individuals had participated in the follow-up program. Of all the participants, 39 were dentate (group A), and 11 had lost all of their teeth (Group B). Table 1 summarizes the demographic data of the subjects in both groups.

**Table 1:** Demographic data of all participants in this study

**Group**	**N**	**Gender N (%)**	**Mean age ± SD**	**Smokers N (%)**	**Systemic disease** **N (%)**	**Periodontal condition at the first visit** **N (%)**
**A**	39	F = 17 (43.6) M =22 (56.4)	52.74 ± 9.30	7 (7.7)	D = 6 (15.38) CD = 3 (7.69)	GMCP = 10 (25.64) GSSP = 29 (74.35)
**B**	11	F = 4 (36.4) M =7 (63.6)	56.18 ± 11.21	4 (36.4)	CD = 5 (45.45)	GMCP = 1 (9.09) GSSP = 8 (72.72) AP = 2 (18.18)

D = Diabetes mellitus type 2 CD = Cardiovascular diseases GMCP = generalized moderate chronic periodontitis GSCP = generalized severe chronic periodontitis AP = aggressive periodontitis

### Groups A and B

**Figure 1:**
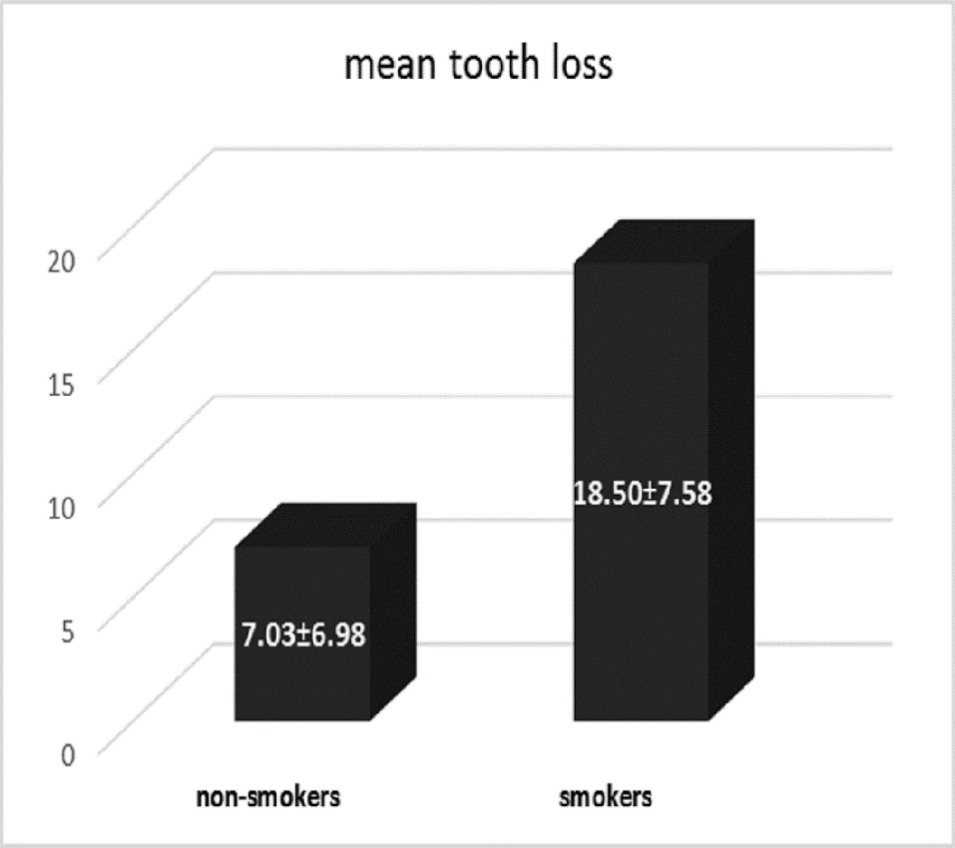
Comparison of tooth loss rate between smokers and non-smokers

The total number of lost teeth in the 50 participants was 339, with a mean of 6.87 teeth. There was no relationship between gender or age and the number of lost teeth (P=0.753 and P=0.642). However, those who had smoked cigarettes had significantly more lost teeth (P=0.001) (Figure 1).

### Group A

The mean PI and BOP were 80.447±27.461 and 67.128±35.724, respectively. Furthermore, 27 participants (69.2%) had 125 new tooth extractions, which was almost equal between the two jaws. The average new tooth loss for individuals in this group was 3.20 teeth.

Table 2 describes the diagnosis of the current status of individuals in group A. There was no significant relationship between PI, BOP, smoking, and systemic disease, on the one hand, and recurrence of periodontitis on the other hand (P=0.090, P=0.157, P=0.320, and P=0.867, respectively). There was no significant relationship between age and periodontitis recurrence, either (P= -0.848). However, the value was negative, suggesting that in younger individuals, the odds of periodontitis recurrence were higher.

**Table 2:** Periodontal conditions of the participants at the first visit and at the time of the study

**First diagnosis**	**Periodontal condition at the time of study N (%)**				
	**Healthy**	**Gingivitis**	**LCP**	**GSCP**	**Total**
**GMCP**	1 (10%)	6 (60%)	3 (30%)	0	10
**GSCP**	0	9 (31%)	8 (27%)	12 (41%)	29
**Total**	1	15	11	12	39

LCP = localized chronic periodontitis GMCP = generalized moderate chronic periodontitis GSCP = generalized severe chronic periodontitis

Nevertheless, there was a significant relationship between initial diagnosis and recurrence rate of periodontitis (P=0.017). Besides, in patients who had been previously diagnosed with generalized severe chronic periodontitis (GSCP) in the initial diagnosis, disease relapse was significantly higher compared to those who had been previously diagnosed with generalized moderate chronic periodontitis (GMCP) (Table 2). There was a significant relationship between disease recurrence and years elapsed since the initial treatment (P=0.027, r=0.353), where the correlation coefficient was positive.

There was no statistically significant relationship between FI and tooth mobility and recurrence of periodontitis (P=0.097 and P=0.748, respectively). However, half of the lost teeth had FI and/or tooth mobility in the first visit.

## Discussion

Although there is a consensus over the significant role of supportive treatment phase in the success of periodontal treatments, patients’ compliance is poor in this regard. Studies have shown that the extent of care and cooperation of patients depends on various factors, including differences in culture, behavior, socioeconomic condition, and personality traits.^17,18^ A precise planning by the dentist or dental hygienist can be useful in developing cooperation.^8^

In this study, which was conducted on participants who did not take part in periodontal maintenance recalls, the recurrence of periodontitis was observed in most individuals (Table 2). Previous studies have also found periodontal disease recurrence in non-compliers.^19-21^ There is a significant relationship between the initial diagnosis and periodontitis recurrence (P=0.017, Table 2). All the patients with recurred GSCP had the same condition at the first visit. Matuliene et al^20^ categorized patients who had adhered to recall intervals during the maintenance period based on BOP percentage, pocket depth >5 mm percentage, the extent of tooth loss, the extent of bone loss, environmental factors such as smoking, and systemic diseases into low, moderate, and high-risk individuals. They observed in 10 years that disease recurrence was higher in high-risk individuals.

In this research, there was a significant relationship between periodontitis relapse and years elapsed since the initial treatment (P=0.027, r=0.353). The correlation coefficient is positive, suggesting that over time, the possibility of disease recurrence increases. Matuliene et al^20^ had also found that, even in patients with regular maintenance, a factor affecting disease recurrence was the duration of the maintenance period (>10 years). Thus, the passage of time can be effective in disease relapse even in patients participating in maintenance periods. The present study showed that disease relapsed in <10 years in patients who did not adhere to the maintenance phase’s regular recall plan.

Nevertheless, in the present study, no significant relationship was found between smoking and systemic disease and the recurrence of periodontitis. However, smokers had lost more teeth (P=0.001). Other studies have found a significant relationship between cigarette smoking or diabetes and the recurrence of periodontitis.^21,22^ They have also reported an increase in tooth loss in smokers.^21,22^ Higher levels of tooth loss in men than in women (approximately two times) have also been reported.^21^ Less cooperation of men for the periodontal supportive treatment phase has been considered as a related factor.^23^ Other factors affecting the tooth loss in the maintenance phase were conservative versus more radical treatment, upper jaw teeth, multi-rooted teeth.^21^ Almost half of the extracted teeth in this study had been diagnosed with mobility or furcation involvement in the first visit of the patient. Nibali et al^24^ reported that the probability of losing molars with furcation involvement in 10–15 years in the maintenance phase is twice as large as that of losing molars without furcation involvement. Nevertheless, they proposed that the molars with furcation involvement should be treated and preserved as much as possible because they found that even molars with furcation involvement of the third degree respond well to the periodontal treatment.^24^ Interestingly, Seirafi et al^1^ did not find any statistically significant difference in periodontal parameters (including tooth loss) of the group with regular follow-up and irregular follow-up, except for BOP.

## Conclusion

The maintenance phase is very crucial in those with a history of periodontitis, in particular for those with more severe disease because the more severe the disease, the higher the chance of disease relapse, thereby increasing the need to participate in recall follow-ups. The probability of disease relapse increases with an increase in the number of years elapsed since the initial periodontal treatment. Thus, it can be suggested that for those with poor cooperation, maintenance follow-ups should at least be set apart with a longer interval to increase the probability of cooperation in these individuals, hence minimizing the chance of relapse.

## Authors’ Contributions

FAM: Conceptualization, data analysis, manuscript preparation and editing. MT: Conceptualization, manuscript editing. FM: data collection, data analysis, review manuscript. SS: data analysis, manuscript editing.

## Competing Interests

The authors declare no conflict(s) of interest related to the publication of this work.

## Ethics Approval

This study was approved by the Ethics Committee of Shahid Beheshti University of Medical Sciences code: IR.SBMU.RIDS.REC.1395.299.
